# Notes and News

**Published:** 1901-11-09

**Authors:** 


### NOTES AND NEWS.

A new bacteriological laboratory has been opened by Sir Frederick Treves at the Royal Infirmary, Bristol.

Whitby has now got a permanent cottage hospital in place of the temporary building which has served the purpose since 1898.

The Council of Charing Cross Hospital have decided to add an ophthalmic department to the hospital. The days and time of attendance for patients will be on Wednesdays and Saturdays at 9 a.m., commencing on Wednesday, November 6th, next.

Mr. Matthew Whiting, of Wandsworth Common, has bequeathed about £120,000 in trust in equal shares for St. Thomas's Hospital, St. George's Hospital, Guy's Hospital, King's College Hospital, the Westminster Hospital, the Middlesex Hospital, the London Hospital, the Royal Free Hospital, St. Mary's Hospital, the Great Northern Hospital, the Brompton Hospital for Consumption and Diseases of the Chest, and the New Hospital for Women, Euston Road.

Many of the improvements which have been effected at Charing Cross Hospital are now completed. A great deal of attention has been devoted to the kitchen department and domestic quarters, and all offices. Not least in importance are the new sanitary annexes to the wards. These and other alterations have cost £12,000. A large expenditure to be faced is that required for the new buildings not yet commenced, though a tender has been accepted and the site is nearly clear.

Sir Hiram Maxim's discovery that it is possible to attract mosquitoes by sound opens up a fresh means of destroying the plague-conveying species. The use of birdlime, respecting which an interesting article appears in this number of The Hospital, might be effective in conjunction with the attractions of sound. Sir Hiram Maxim accidentally alighted on the pitch of sound which attracted the male mosquito only. A still more valuable discovery would be of the sound which attracts the female insect, but this will probably follow.

Whilst in England a tendency to laxity in medical etiquette is gradually gaining ground, in Germany efforts are being put forth to render the observance of the unwritten code stricter. In Berlin a Medical Court of Honour has been formed, and it has recently made decisions condemning medical men guilty of puffing self advertisement.

Strenuous efforts are being made in Glasgow to prevent the plague from spreading. The Central Station Hotel, where the cases occurred, has been closed and means taken to exterminate all rats and disinfect the premises. The contacts were all placed in quarantine. Glasgow has been declared an infected port. In Liverpool no further developments have taken place.

The new ward for women at the Cardiff Infirmary is now open. Thirteen beds are available, each being maintained from a separate source. Efforts are being made to endow other beds, and three more are expected to be shortly available. This method of fixing interest and responsibility is a very good one, and is much more likely to stimulate emulation than the publication of a list of subscriptions to be allocated to the general needs of an infirmary.

Small-pox continues to make itself a nuisance in London. Fortunately the scare about it spreads even more quickly than the disease, and many people are being vaccinated. It seems a pity that even in the present day "the people" refuse to be satisfied with anything short of human sacrifice. Small-pox, however, is a great teacher and is grinding in its lesson; these martyrs have not died entirely without avail, for the people at last are rushing to vaccination.

In response to the appeal issued by the Organising Committee of the Prince of Wales's Hospital Fund for London to factories, workshops, etc., the following amounts have been received:—The employees of the British Asbestos Company, Limited, £1 13s. 8d.; the employees of the Tandem Smelting Syndicate, Limited, £1 10s.; the employees of Messrs. Elphinstone and Co., 15s. Further contributions have been received from the Fels-Naphtha Soap Company and Mr. S. H. Benson, £12 18s. 7d.; the Orchestrelle Company (per Mr. A. J. Mason), £10 10s.; Messrs. Bewlay and Co., Limited, £2 2s.; Mr. A. Wormull, £1 1s.; the Hoyt Metal Company of Great Britain, £1; Miss Ellen Collett, 5s.

Alderman Treloar asks us to bring before the notice of our readers his annual entertainment to poor children in the Guildhall of the City of London, and the distribution of Christmas hampers to 4,000 or 5,000 little cripples. The Keeper of the Privy Purse has written to Alderman Treloar, "I have the pleasure to inform you that His Majesty will be very happy to continue his support to the Christmas and New Year's entertainment which you so kindly organise every year for the benefit of the Ragged School children of London." It is therefore with confidence that I again appeal to you to assist me in raising the necessary funds. Last year £1,434 1s. 6d. was collected, but year by year the number of crippled children registered increases, and the difficulty of selecting the recipients for the hamper becomes greater. Donations should be addressed to 69 Ludgate Hill, London, E.C.

